# The Efficacy of Ginsenoside Rg3 Combined with First-line Chemotherapy in the Treatment of Advanced Non-Small Cell Lung Cancer in China: A Systematic Review and Meta-Analysis of Randomized Clinical Trials

**DOI:** 10.3389/fphar.2020.630825

**Published:** 2021-03-18

**Authors:** Ze Peng, Wen Wen Wu, Ping Yi

**Affiliations:** ^1^ Institute of Integrated Traditional Chinese and Western Medicine, Tongji Hospital, Tongji Medical College, Huazhong University of Science and Technology, Wuhan, China; ^2^ West China School of Medicine, Sichuan University, Chengdu, China; ^3^ Department of Integrated Traditional Chinese and Western Medicine, Tongji Hospital, Tongji Medical College, Huazhong University of Science and Technology, Wuhan, China

**Keywords:** Ginsenoside Rg3, advanced non-small cell lung cancer, first-line chemotherapy, meta-analysis, systematic review

## Abstract

**Background:** For advanced non-small cell lung cancer (NSCLC) patients, first-line chemotherapy is the main treatment in the clinic despite its efficacy is limited and adverse effects are always inescapable. Ginsenoside Rg3, an anti-cancer active ingredient by suppressing angiogenesis, has been increasingly widely used as an adjuvant in first-line chemotherapy for advanced NSCLC to optimize treatment in China. However, no comprehensive meta-analyses have been conducted to estimate the efficacy and safety of the therapy combining ginsenoside Rg3 and first-line chemotherapy in advanced NSCLC patients.

**Methods:** Randomized controlled trails using a combination of first-line chemotherapy and ginsenoside Rg3 for advanced NSCLC patients were searched and selected from six databases. The Cochrane Risk of Bias tool was used to assessed the quality of these selected original researches. And we used Review Manager 5.3 and STATA to analyze the data.

**Results:** Twenty-two RCTs that matched our selection criteria with a number of 2202 patients were included in our review. The results showed that compared with first-line chemotherapy alone, the combination of ginsenoside Rg3 and first-line chemotherapy could better improve the objective response rate (ORR) (RR [95% CI], 1.44 [1.27, 1.63], *p* < 0.00001 ), the disease control rate (DCR) (RR [95% CI], 1.24 [1.12, 1.38], *p* < 0.0001), karnofsky performance status (KPS) (RR [95% CI], 1.62 [1.42, 1.84], *p* < 0.00001), one-year survival rate (RR [95% CI], 1.49 [1.08, 2.06], *p* = 0.01), two-year survival rate (RR [95% CI], 6.22 [1.68, 22.95], *p* = 0.006), weight change (RR [95% CI], 1.31 [1.04, 1.66], *p* = 0.02), and higher reduce the VEGF levels (RR [95% CI], -2.21 [-4.03, -0.38], *p* = 0.02), the incidence of gastrointestinal reactions (RR [95% CI], 0.66 [0.47, 0.93], *p* = 0.02) and bone marrow suppression (RR [95% CI], 0.43 [0.30, 0.61], *p* < 0.00001).

**Conclusion:** Ginsenoside Rg3 can enhance drug efficacy and reduce drug-induced toxicity from chemotherapy. These findings provide helpful information for clinicians indicating that a therapy combined of ginsenoside Rg3 and first-line chemotherapy may be used to optimal the treatment of advanced NSCLC.

## Introduction

As a serious health issue all over the world, lung cancer has the highest morbidity and mortality in all cancers (Siegel et al., 2020). Approximately 85% of patients with lung cancer have a group of histological subtypes that are collectively known as non-small cell lung cancer (NSCLC) (Herbst et al.,2018). Since the majority of patients with NSCLC are diagnosed at an advanced stage (III or IV) when the cancer cells have usually metastasized (Du and Morgensztern 2015), most of them unfortunately lose the opportunity for surgical treatment. Only 18% of patients with advanced NSCLC undergo surgery, and nearly 62% advanced patients with NSCLC are treated with radiation treatment and systemic treatment including chemotherapy, targeted therapy and immunotherapy to improve long-term survival rate (Miller et al., 2019). Although in the past several years, great progress has been significantly made in molecularly targeted therapy and immunotherapy, first-line chemotherapy remains a mainstay in the therapeutic management of NSCLC (Rossi and Di, 2016; Nasim et al., 2019). Particularly, platinum-based regimens are the most active combinations in clinical practice and show a meaningful clinical benefit for advanced NSCLC patients (Watanabe et al., 2017; Herbst et al.,2018).

However, although platinum-based regimens show several benefits for patients with advanced NSCLC, only a minority of patients indeed achieve an objective treatment response and the 5-year survival rate is still less than 20% (Rose et al., 2014). Moreover, it cannot be ignored that chemotherapy drugs often lead to severe side effects, such as bone marrow suppression, serious gastrointestinal reaction and liver abnormalities (Islam et al.,2019; von Plessen, 2011). And another limitation of first-line chemotherapy is the increasing ineffectiveness of chemotherapy caused by drug resistance. It’s reported that the drug resistance induced by platinum-based chemotherapies can be as high as 70% in advanced NSCLC patients (Rossi and Di Maio, 2016; Zhao and Chen, 2020). Therefore, looking for optimal therapy which can improve the efficacy of chemotherapy and reduce the incidence of side effects is of great necessity.

In recent years, traditional Chinese medicine (TCM) has played an increasingly important role in anti-cancer for its efficacy and security (Xiang et al., 2019). The combination of Chinese and Western therapy in anti-cancer treatment has become a hot trend all over the world (Liao et al., 2017; So et al., 2019). Ginseng, as a famous Chinese herbal medicine, has a medicinal history of four thousand years in China and is well known as ‘the king of herbs’ (Wang and Jin, 2018). Ginsenoside Rg3, a naturally active ingredient extracted from ginseng leachate, has been showed to possess anti-cancer in various tumors, especially in advanced NSCLC (Li Y et al., 2016). The therapeutic effects of ginsenoside Rg3 include inducing tumor apoptosis, inhibiting tumor metastasis, suppressing tumor angiogenesis and reversing drug resistance (Luo et al., 2019; Nakhjavani et al., 2020). In addition, there is a synergistic effect when ginsenoside Rg3 is used in combination with chemotherapy drugs (Sun et al., 2017; Wang and Jin, 2018; Zhao and Chen, 2020). Nowadays, an increasing number of studies have indicated that ginsenoside Rg3 may be a widely applied natural medicine in the treatment of NSCLC (Gao and Liu, 2019) and its combination with first-line chemotherapy drugs may brings great promise to the management of advanced NSCLC.

At present, some clinical trials of ginsenoside Rg3 combined with first-line chemotherapy on NSCLC have been conducted in China. However, no comprehensive meta-analyses have been conducted to estimate the efficacy and safety of this combination therapy. The purpose of this systematic review and meta-analysis is to evaluate the efficacy and safety of the therapy combining ginsenoside Rg3 and first-line chemotherapy in advanced NSCLC.

## Materials and Methods

### Search Strategy

A systematic literature search for RCTs testing the combination of ginsenoside Rg3 and first-line chemotherapy in advanced NSCLC published from the inception of each database to October 2020 was conducted without language restrictions in six databases, including PubMed, Web of Science, the Cochrane Library, Wan Fang Database, Chinese VIP Information (VIP) and China National Knowledge Infrastructure (CNKI).

A free term strategy was used, for Chinese databases, the following terms were used in combined ways: “Renshen zao gan Rg3 or Shenyi jiaonang” and “feixiao xibao feiai”; for English databases, we used text terms including “ginsenoside Rg3 or Shenyi capsule” and “NSCLC”.

### Selection Criteria

The inclusion criteria were as follows: 1) patients: age of ≥18 years, histological or cytological confirmation of advanced NSCLC. 2) interventions: the control group were treated with first-line chemotherapy and the combination of ginsenoside Rg3 and first-line chemotherapy was conducted in the experiment group relatively. 3) the outcome should include at least one of the following indicators: objective response rate, disease control rate, Karnofsky`s performance score (KPS) and adverse effects. 4) study design: randomized clinical trials (RCTs).

The exclusion criteria were as follows: 1) non-RCTs including case reports, reviews, animal or cell studies and studies without a control group. 2) patients treated with other therapies, expect for ginsenoside Rg3 and chemotherapy. 3) patients with small cell lung cancer (SCLC) or other serious diseases. 4) early NSCLC.

### Data Extraction

Two independent reviewers (Ze Peng and Wen Wen Wu) extracted the data according to the inclusion criteria. The characteristics of the data consisted of the author, publication year, the number and sex of participants, interventions, treatment cycles, the stage of NSCLC and outcomes. Where outcomes were ambiguous or missing in an article, the decision to retrieve from that article was resolved by consensus.

### Methodological Quality

The Cochrane Risk of Bias tool was used to assessed the quality of the literature by two reviewers (Ze Peng and Wen Wen Wu) separately. The assessed items included: 1) random sequence and allocation concealment; 2) blinding of participants and personnel; 3) blinding of outcome assessment; 4) incomplete outcome data; 5) selective reporting; 6) other bias. For any disagreement, the risk of bias was discussed by consensus.

### Data Synthesis and Analysis

We performed the analysis by using Review Manager (ver. 5.3) and STATA (ver.14) software. For binary variables, the risk ratio (RR) with 95% confidence interval (CI) was used to evaluate the pool effects. *p* value <0.05 was considered statistically significant. We used Chi-squared and I^2^ tests to evaluate the heterogeneity of all studies included. The fixed-effect model was selected for analysis with *p* > 0.05 or I^2^ < 50%; otherwise, we choose the random-effect model. And we assessed the sensitivity analysis to state the effect of changing the study model on the pooled analysis results. Begg’s tests were carried out to determine the publication biases and *p* value >0.05 was considered as no publication bias.

## Results

### Study Inclusions

**FIGURE 1:**
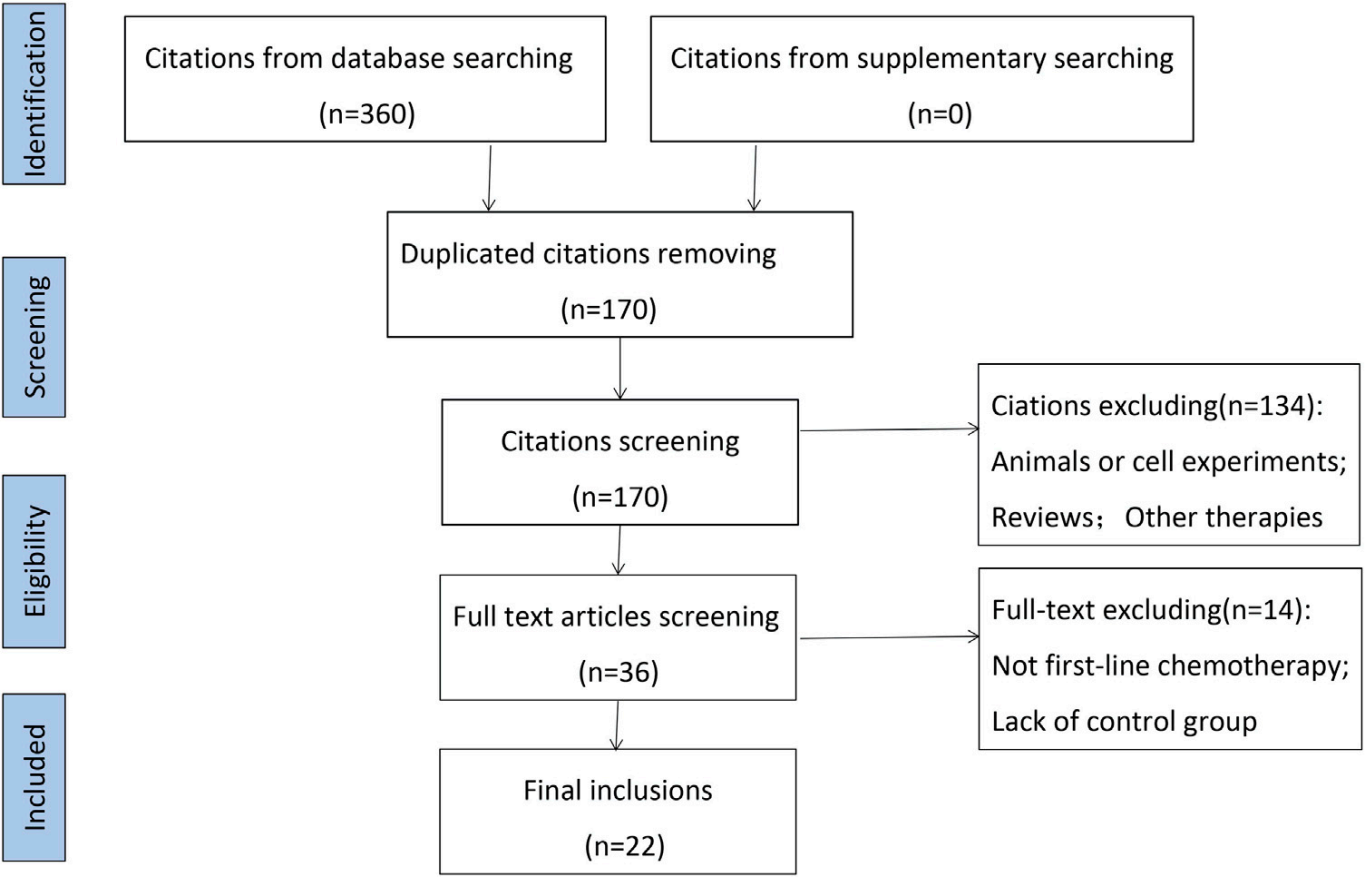
Flow chart of study selection process.

A total of 360 studies were identified through systematic search, which included 190 repeated records (Figure 1). After a first screening, 36 were included by browsing the titles and abstracts of the remaining 170 studies. Then the remaining studies were further carefully scrutinized, and 134 articles were eliminated due to animals or cell experiments (*n* = 19), review (*n* = 16) and other therapies consisting of radiotherapy, postoperative lung cancer, and maintenance therapy (*n* = 99). Finally, excluding the studies with no control group or non-first-line chemotherapy regimens, 22 articles were included in this systematic analysis (Chen and Li 2012; Chen et al., 2014; Du 2014; Li and Bai 2017; Li et al., 2012; Liang and Han 2016; Lin et al., 2014; Liu et al., 2007; Liu et al. 2009; Liu et al. 2015; Pan and Wu, 2016; Pan et al., 2019; Pang, 2012; Qi and Zhang 2011; Shen et al., 2018; Sun et al., 2006; Tu, 2008; Wang et al., 2020; Wang et al., 2011; Wang et al., 2017; Zhang et al., 2020; Zhang et al., 2018).

### Characteristics of the Studies

Twenty-two studies with 2202 patients were included in our review. All RCTs originated from China and were published in Chinese journals. The year of publication was between 2006 and 2020, with 19 studies from the last decade. The patients’ characteristics of included studies included age, gender, clinical stage, chemotherapy regimen, and treatment indicators and were summarized in Table 1.

**TABLE 1:** Characteristics of RCTs included in the study.

Study	Sample size(E/C)	Age	Sex(Man/Feman)	Clinical stage	Pathological type	Experiment Group(E)	Control Group(C)	Treatment Cycle	First Treatment	Outcome
Chen and Li (2012)	35/35	55.5/60.5	24/11;	III 14,IV 21;	S20,A15;	Rg3 20 mg	GP	6-9 weeks	NO	Efficac ( RECIST ); Adverse reactions; KPS
			22/13	III 13,IV 22	S18,A17	po.bid +C				
Chen et al. (2014)	34/34	4 1 ∼ 7 3	39/29	IIIB-IV	A26,S21,AC18,B3	Rg3 20 mg	TP	12 weeks	No	Efficacy(RECIST); Adverse reactions; Immune index
						po.bid +C				
Du (2014)	30/30	40.2 ± 3.6	31/29	advanced	NSCLC	Rg3 20 mg	TP	6 weeks	No	Efficacy(RECIST);KPS
						po.bid +C				
Li and Bai (2017)	90/90	57 ± 1. 3/58 ± 1. 0	49/41;	advanced	NSCLC	Rg3 20 mg	GP	9 weeks	No	Efficacy(RECIST); PFS,OS; Adverse reactions
			49/41			po.bid +C				
Li et al. (2012)	39/38	-	-	advanced	A23,S14,B2;A20,S16,B2	Rg3 20 mg	GP	6 weeks	No	Efficacy(RECIST); KPS;Adverse reactions PFS;one-year survival rate
						po.bid +C				
Liang and Han (2016)	47/46	67.47 ± 7.74/66. 32 ± 6. 21	34/13;	IIIA-IV	NSCLC	Rg3 20 mg	GP	12 weeks	No	KPS;Adverse reactions
			31/15			po.bid +C				
Lin et al. (2014)	33/25	65-85	32/26	IIIA6,IIIB6,IV46	A44,S8,P6	Rg3 20 mg	PC	6 weeks	No	Efficacy(RECIST); KPS;Adverse reactions
						po.bid +C				
Liu et al. (2007)	35/35	35-70	43/27	IIIB-IV	S26,A40,B4	Rg3 20 mg	NP	6 weeks	Yes	Efficacy(WHO); KPS;Adverse reactions;Immune index
						po.bid +C				
Liu et al. (2009)	34/30	43-75/31-66	26/8;19/11	IIIB22,IV12/IIIB26,IV4	A21,S9,AC4/A21,S6,AC2,B1	Rg3 20 mg	NP	6 weeks	No	Efficacy(WHO);Adverse reactions;one-year survival rate
						po.bid +C				
Liu et al. (2015)	60/60	52.5 ± 2.0/54.6 ± 2.1	46/14;35/25	III37,IV23/III29,IV31	A19,S41/A13,S46	Rg3 20 mg	NP	-	No	Efficacy(unclear);Adverse reactions
						po.bid +C				
Pan and Wu (2016)	24/24	71.5/71	16/8;15/9	advanced	unclear	Rg3 20 mg	TP	6 weeks	No	Efficacy(RECIST); KPS;Adverse reactions
						po.bid +C				
Pan et al. (2019)	103/104	60.6 ± 10.4/62.5 ± 11.9	53/50;51/53	III-IV	unclear	Rg3 20 mg	TP	9 weeks	Yes	Efficacy(RECIST); KPS;Immune index
						po.bid +C				
Pang (2012)	22/21	63.95	26/17	IIIB13,IV30	A26,S18	Rg3 20 mg	GP/PC/TP	6 weeks	Yes	Efficacy(RECIST); KPS;Adverse reactions
						po.bid +C				
Qi and Zhang (2011)	35/35	37-70	48/22	advanced	A40,S26,B4	Rg3 20 mg	GP	6 weeks	Yes	Efficacy(unclear); KPS;Adverse reactions
						po.bid +C				
Shen et al. (2018)	25/27	66-78/66-77	14/11;17/10	IIIB-IV	A15,S10/A19,S8	Rg3 20 mg	GP/PC	6 weeks	Yes	Efficacy(WHO); Adverse reactions;one-year survival rate
						po.bid +C				
Sun et al. (2006)	51/50	59.54/57.44	40/14;39/22	III21,IV33/III24,IV37	S16,A27,AC6,05;S13,A44,AC2,O2	Rg3 20 mg	NP	6 weeks	No	Efficacy(WHO); Adverse reactions; PFS
						po.bid +C				
Tu (2018)	21/20	-	13/8;11/9	III7,IV14;III8,IV12	A10,S7,04;A9,S9,02	Rg3 20 mg	TP	6 weeks	Yes	Efficacy(RECIST); KPS;VEGF
						po.bid +C				
Wang et al. (2020)	39/39	54.9 ± 8.1/55.6 ± 7.8	21/18;22/17	IIIB18,IV21;IIIB19,IV20	A20,S19;A21,S18	Rg3 20 mg	GP/PC	6 weeks	No	Efficacy(WHO); Adverse reactions
						po.bid +C				
Wang et al. (2011)	59/58	53	74/43	IIIB-IV	A31,S28/A37,S21	Rg3 20 mg	GP/NP	-	Yes	VEGF;KPS;Adverse reactions
						po.bid +C				
Wang et al. (2017)	45/44	58. 95	52/37	IIIb41,IV48	A58,S31	Rg3 20 mg	TP/PC/GP/NP	6 weeks	No	Efficacy(unclear);KPS;Adverse reactions
						po.bid +C				
Zhang et al. (2020)	41/41	71. 34 ± 4. 25/71. 52 ± 3. 65	27/14;26/15	IIIb18,IV23/IIIb17,IV24	UNCLEAR	Rg3 20 mg	GP	12 weeks	No	Efficacy(WHO);KPS;Immune index; Adverse reactions
						po.bid +C				
Zhang et al. (2018)	199/215	61.16 ± 10.41/60.76 ± 10.39	128/71;161,64	IIIA25,IIIB74,IV100/IIIA20,IIIB73,IV122	A114,S65,B3,017/A121,S72,B3,O19	Rg3 20 mg	NP/TP	≥6 weeks	Yes	Adverse reactions
						po.bid +C				

S, squamous cell carcinoma; A, adenocarcinoma; AC, adenosquamous carcinoma; B, large cell carcinoma; O, poorly differentiated carcinoma; GP, gemcitabine; TP, PTX; PC, pemetrexeddisodium; NP, navelbine.

### Quality Assessment

**FIGURE 2:**
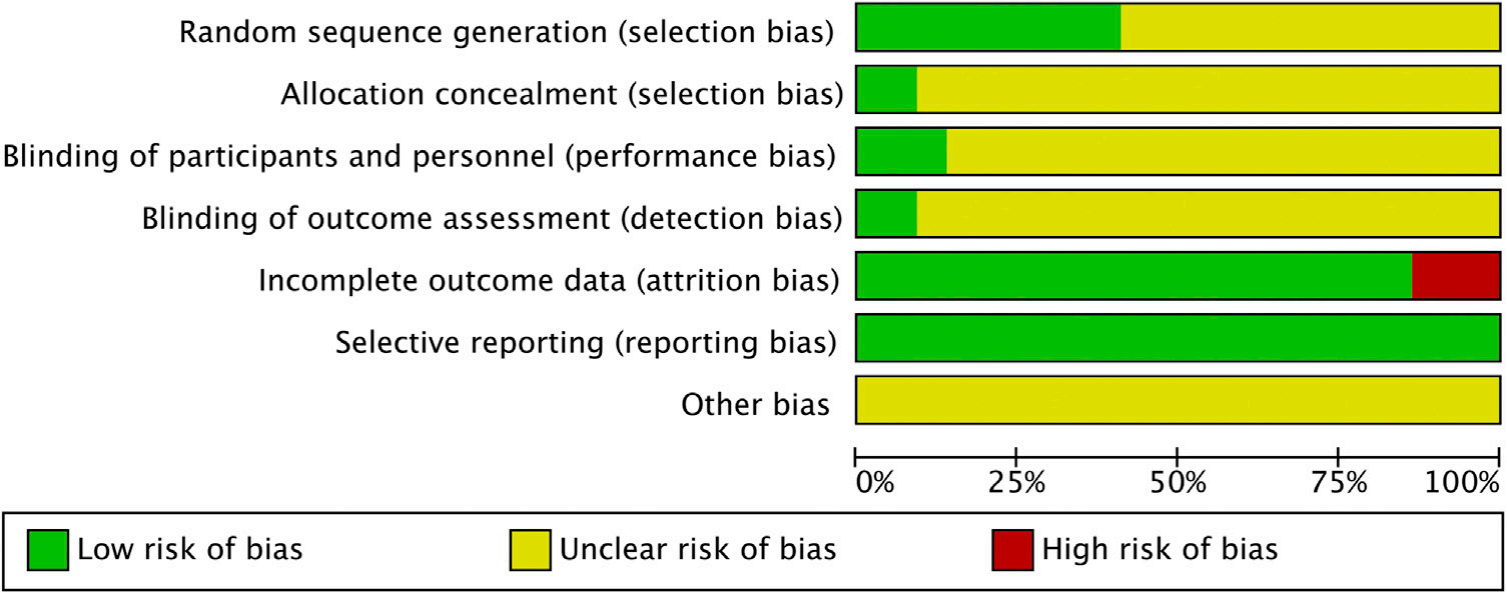
Risk of bias.

**FIGURE 3:**
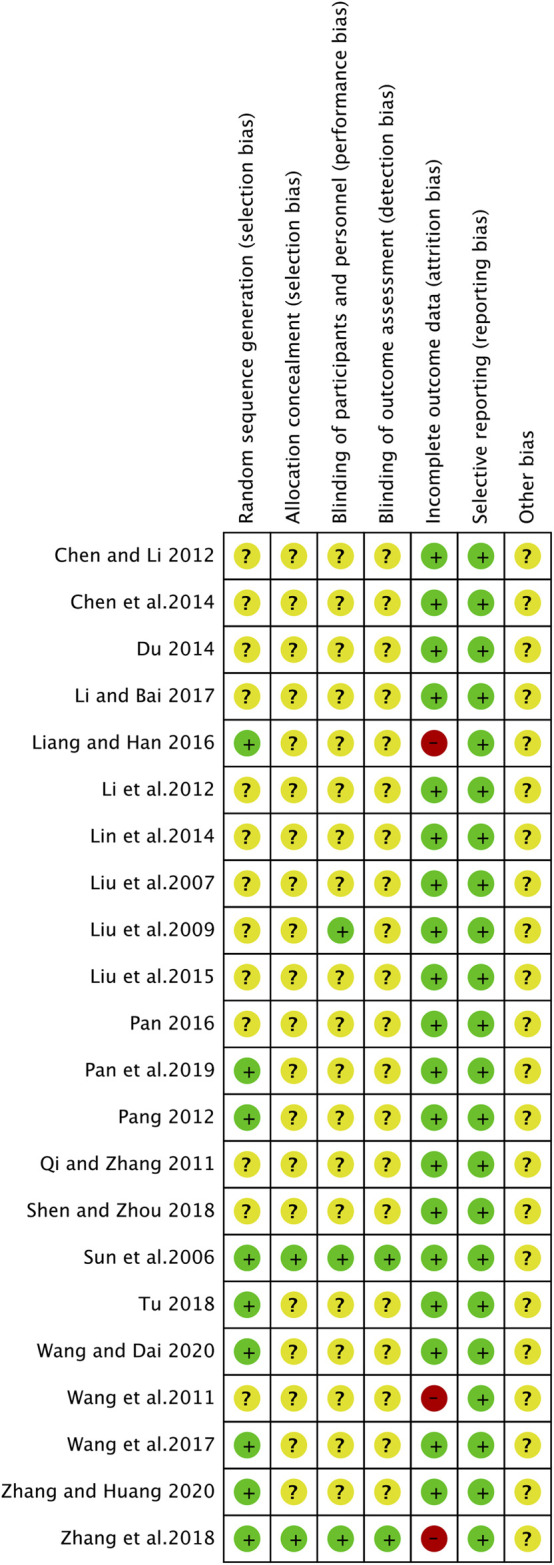
Risk of bias summary.

Figure 2 showed our assessment of the bias risk of the included studies by Review Manger 5. All included studies described the process of random sequence generation. However, only 9 of the 22 studies described the detailed process of avoiding selection bias and just two studies reported the allocation concealment and blinding of outcome assessment in detail. As for the performance bias, only three studies mentioned and the rest were not. Among the included studies, three articles were identified as high risk of reporting bias for outcome because the disease control rate was not reported. Nevertheless, they included other important indicators to evaluate the efficacy and safety of ginsenoside Rg3 in combination with first-line chemotherapy in advanced NSCLC, such as KPS, weight change, and side effects. Therefore, after our discussion, we decided to include these three studies in this analysis. The results of each study were reported faithfully, therefore we considered all studies to be free of reporting bias (Figures 2, 3).

### Tumor Response

**FIGURE 4:**
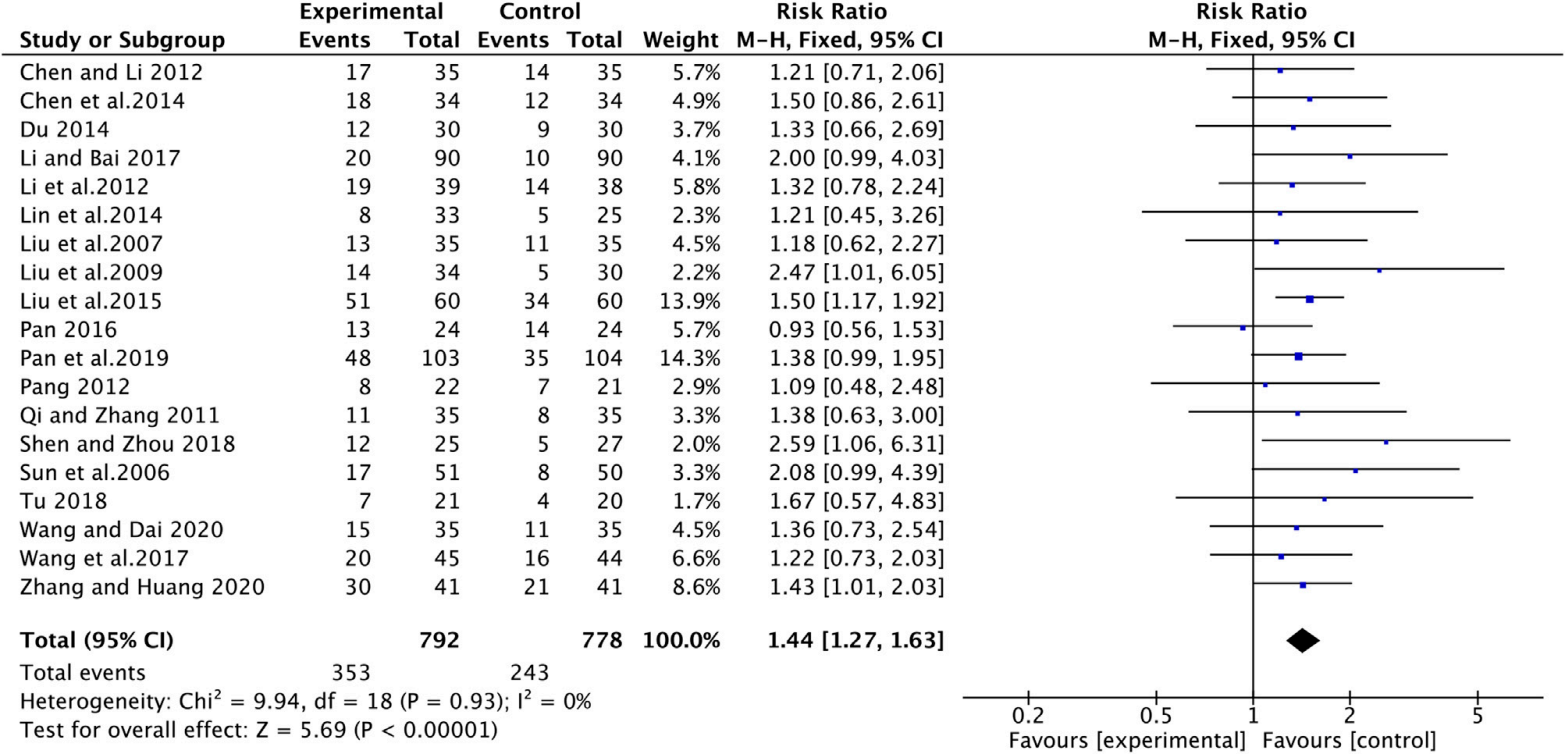
The pooled effects of ginsenosides Rg3-containing chemotherapy on objective response rate.

**FIGURE 5:**
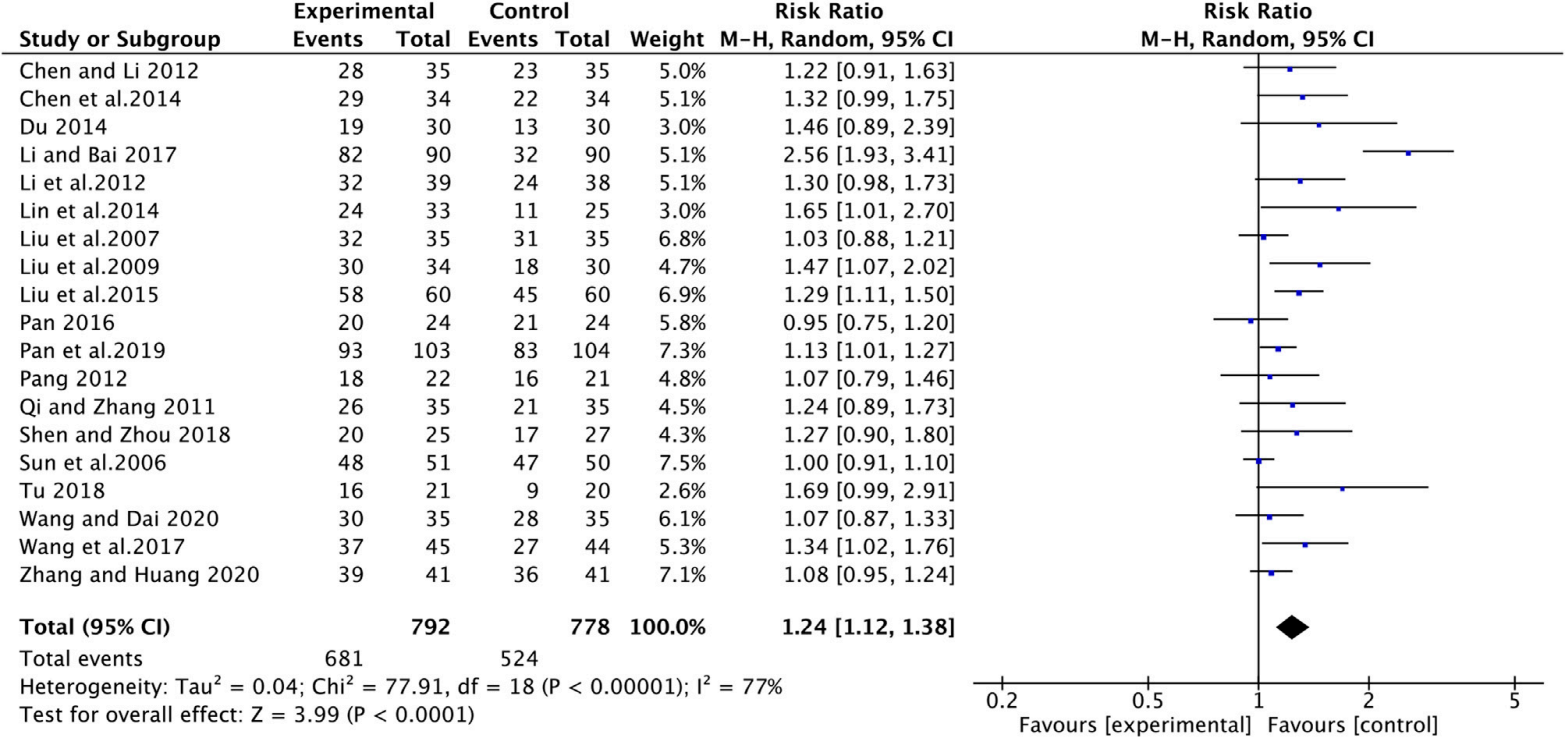
The pooled effects of ginsenosides Rg3-containing chemotherapy on disease control rate.

Nineteen studies with 1470 advanced NSCLC participants recorded the short-term treatment efficiency of ginsenoside Rg3 combined with first-line chemotherapy. The overall heterogeneity of the meta-analysis showed that the included studies had clinical and statistical homogeneity, so a fixed-effect model was chosen. As illustrated in Figure 4, the experimental group had a higher objective response rate (ORR) than the control group (RR [95% CI], 1.44 [1.27, 1.63], *p* < 0.00001). For disease control rate (DCR), heterogeneity test (I^2^ = 77%) suggested that there was considerable heterogeneity, so we turned to a random effect model. Compared with chemotherapy alone, the combine of ginsenoside Rg3 and chemotherapy had a better effect on DCR (RR [95% CI], 1.24 [1.12, 1.38], *p* < 0.0001) (Figure 5). Subsequently, we performed regression analysis and subgroup analysis, suggesting that there was no difference in year of publication, evaluation criteria, chemotherapy drugs and first treatment (Supplementary Figures S1, S2).

### Quality of Life

**FIGURE 6:**
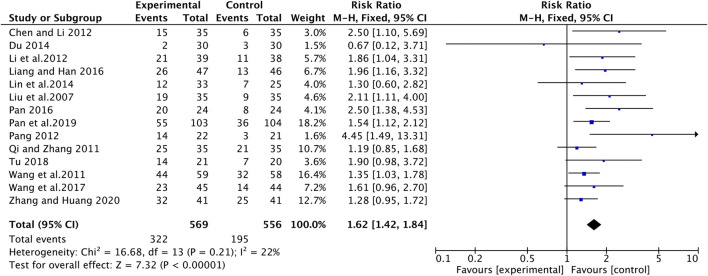
The pooled effects of ginsenosides Rg3-containing chemotherapy on KPS.

Karnofsky performance status (KPS) score was used to evaluate quality of life. Fourteen of the twenty-two studies evaluated the effect of ginsenoside Rg3 combined with chemotherapy on quality of life in patients with advanced NSCLC (Figure 6). An increased of 10 or more in KPS score after treatment was considered as a significant improvement in quality of life, otherwise, it was considered as a stable or even deteriorating quality of life. There was no clinically and statistically significant heterogeneity in these trials. For KPS increase rate, the experimental group was significantly higher than the control group (RR [95% CI], 1.62 [1.42, 1.84], *p* < 0.00001). As for KPS stability rate, the experimental group was higher when compared with the control group (RR [95% CI], 1.21 [1.08, 1.36], *p* = 0.001) (Supplementary Figure S3).

### Year Survival Rate

**FIGURE 7:**
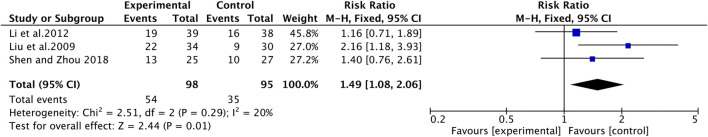
The pooled effects of ginsenosides Rg3-containing chemotherapy on one-year survival rate.

**FIGURE 8:**
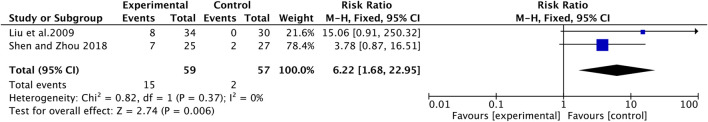
The pooled effects of ginsenosides Rg3-containing chemotherapy on two-year survival rate.

For patients with advanced NSCLC, survival rate is an important parameter to evaluate the therapeutic effect. A fixed-effect model was chosen because of the low heterogeneity. Three articles reported one-year survival rate, the results showed that ginsenoside Rg3 combined with chemotherapy had a higher one-year survival rate than chemotherapy alone (RR [95% CI], 1.49 [1.08, 2.06], *p* = 0.01) (Figure 7). Two studies reported two-year survival rate, the results showed that ginsenoside Rg3 combined with chemotherapy group had a higher two-year survival rate than chemotherapy alone group (RR [95% CI], 6.22 [1.68, 22.95], *p* = 0.006) (Figure 8).

### Weight Change

A total number of five studies reported changes in body weight before and after treatment, and weight gain of ≥1 kg was defined as weight improvement. Compared with the control group, the experimental group had a higher rate of weight improvement. (RR [95% CI], 1.31 [1.04, 1.66], *p* = 0.02) (Supplementary Figure S4).

### VEGF Leaves

Four articles reported the changes in serum VEGF levels before and after chemotherapy. Because of the high heterogeneity, we chose the random effect model. The experimental group could reduce the level of VEGF more effectively after treatment compared to the control group (RR [95% CI], −2.21 [−4.03, −0.38], *p* = 0.02) (Supplementary Figure S5).

### Side Effects

**FIGURE 9:**
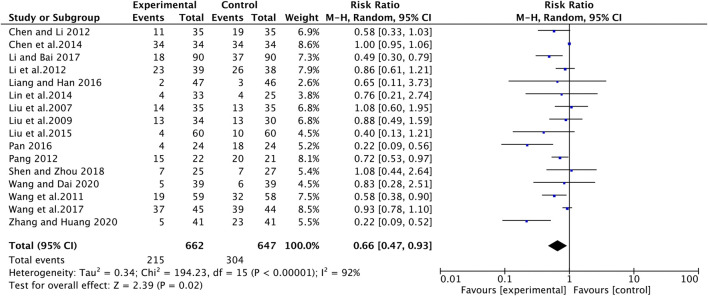
The pooled effects of ginsenosides Rg3-containing chemotherapy on gastrointestinal reactions.

The side effects of chemotherapy mainly include gastrointestinal reactions, liver and kidney injury, bone marrow suppression, and hematological toxicity. The incidence of liver and kidney dysfunction (RR [95% CI], 0.72 [0.52, 1.00], *p* = 0.05) and hematotoxicity (thrombocytopenia: RR [95% CI], 0.64 [0.33, 1.22], *p* = 0.17; leukopenia: RR [95% CI], 0.82 [0.66, 1.02], *p* = 0.07; hemoglobin reduction: RR [95% CI], 0.84 [0.60, 1.17], *p* = 0.29) in the experimental group was not statistically different compared with the control group (Supplementary Figures S6–S9). The experimental group had a lower incidence of gastrointestinal reactions compared to the control group (RR [95% CI], 0.66 [0.47, 0.93], *p* = 0.02) (Figure 9). Although some studies influenced the overall heterogeneity of this metric (I^2^ = 92%; *p* < 0.00001), the overall effects remained largely significant. In addition, six studies reported the occurrence of myelosuppression and showed that the experimental group had a lower incidence of myelosuppression than the control group (RR [95% CI], 0.43 [0.30, 0.61], *p* < 0.00001) , with no heterogeneity (I^2^ = 0%; *p* = 0.57) (Supplementary Figure S10).

According to WHO toxicity classification criteria, grade III-IV was defined as serious adverse reactions. The incidence of leukopenia in the experimental group was lower than that in the control group among the serious adverse reactions (RR [95% CI], 0.48 [0.34, 0.67], *p* < 0.001) (Supplementary Figure S11), and the remaining serious adverse reactions were not statistically different.

### Publication Bias and Sensitivity Analysis

**FIGURE 10:**
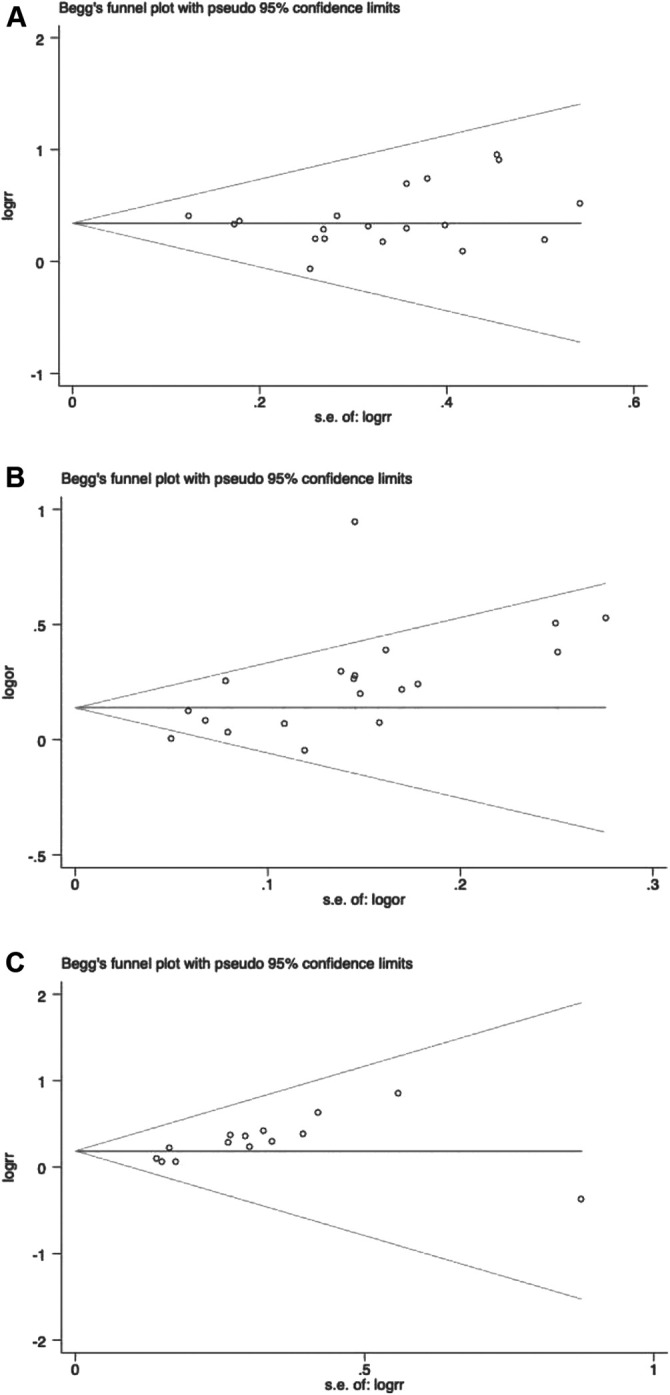
Begg`s regression analyses for publication bias. **(A)** Objective response rate; **(B)** Disease control rate; **(C)** KPS.

**FIGURE 11:**
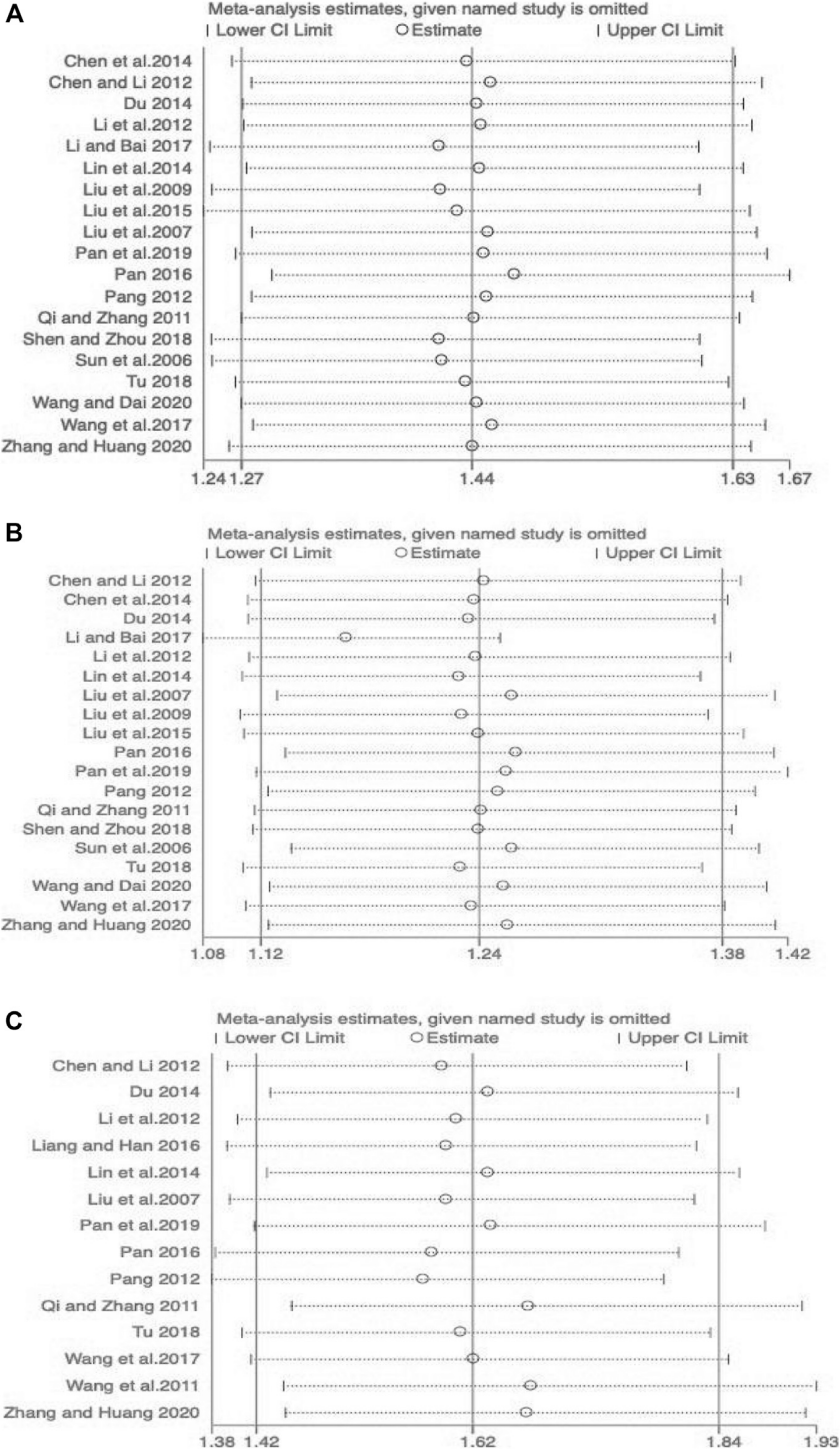
Sensitivity analysis plots. **(A)** Objective response rate; **(B)** Disease control rate; **(C)** KPS.

We performed publication bias analysis on the main parameters and the results showed that publication bias may have an impact on disease control rate and KPS (ORR: *p* = 0.555; DCR: *p* = 0.008; KPS: *p* = 0.015) (Figure 10). We conducted sensitivity analyses of the key effect indicators, including objective response rate, disease control rate, and KPS, and the results showed all to be authentic, verifiable, and of good stability (Figure 11).

## Discussion

In recent years, the combination of ginsenoside Rg3 and chemotherapy has been increasingly proposed and conducted in advanced NSCLC. This systematic review and meta-analysis is the latest to evaluate the efficacy and safety of the therapy combining ginsenoside Rg3 and first-line chemotherapy in advanced NSCLC. The results showed that ginsenoside Rg3 in combination with first-line chemotherapy resulted in better objective response rate, disease control rate, KPS score and one-/two-year survival rate, higher increases in patients weight, and higher reduction in VEGF levels and side effects compared with chemotherapy alone.

According to the Response Evaluation Criteria in Solid Tumors (RECIST), assessment of the change in tumour burden is an important feature of the clinical evaluation of cancer therapeutics: both tumour shrinkage (objective response) and disease progression are useful endpoints in clinical trials (Eisenhauer et al., 2009). ORR was defined as complete response (CR) or partial response (PR). Disease control rate (DCR)was defined as CR or PR in all patients or stable disease (SD) in patients with progressive disease (PD) at the treatment of chemotherapy. At the current time ORR carries with it a body of evidence greater than for any other biomarker supporting its utility as a measure of promising treatment effect in clinical trails.

In our study, 19 studies reported ORR and DCR. Consistent with previous meta-analysis, the results suggested that ginsenoside Rg3 in combination with chemotherapy had a significant advantage in improving ORR (RR 1.44) and DCR (RR 1.23). Subsequently, we classified the patients included in the study as whether they were receiving antineoplastic therapy for the first time. Subgroup analysis showed no statistical difference between the two groups, indicating that chemotherapy containing ginsenosides was significantly beneficial in improving ORR, regardless of whether the patient had received prior anticancer treatment. In addition, there are four main first-line chemotherapy drugs in clinical practice, namely gemcitabine (GP), paclitaxel (TP), norvinblastine (NP), and pemetrexide (PC). The results of subgroup analysis showed that ginsenoside combination chemotherapy improved ORR independent of the type of chemotherapeutic agent. The above results suggested us that ginsenoside Rg3 a broad application prospect in the clinical field.

KPS is the Karnofsky performance status scoring standard. The higher the score, the better you are and the more you can tolerate the side effects of treatment, therefore, NSCLC patients are likely to receive complete chemotherapy (Terret et al., 2011). Adding ginsenoside Rg3 to chemotherapy could effectively improve the quality of life of patients with advanced NSCLC and reduce the suffering caused by the disease and chemotherapy (Lu et al., 2008). Previous meta-analysis had demonstrated that ginsenoside combined with chemotherapy could significantly increase KPS in patients with NSCLC (Xu et al., 2016). In our analysis, summary estimates of 14 trials also showed a significant improvement of KPS in the treatment group compared with control group, the improvement was statistically significant. In addition, for the first time, we included weight change as an indicator, which is also important for assessing quality of life. Our results showed that ginsenoside Rg3 combined with chemotherapy was effective in increasing the weight of NSCLC patients (*p* = 0.02).

Annual survival rate is an important parameter to evaluate the prognosis of patients with advanced cancer. Ginseng itself has anti-cancer properties and health benefits, which has been used for centuries in Oriental medicine as a panacea to promote longevity (Kiefer and Pantuso, 2003; Wang et al., 2016). In our analysis, one-year survival and two-year survival rates were reported for the first time. The results showed that ginsenoside Rg3 combined with chemotherapy significantly improved survival time in patients with advanced NSCLC compared with chemotherapy alone. Unfortunately, the credibility of the evidence was relatively low due to the paucity of literature, and more data were needed to support this conclusion.

The vascular endothelial growth factor A (usually referred to as VEGF) play a central role in angiogenesis, promoting endothelial cell proliferation, migration and invasion. Recent evidence shows that VEGF directly targets tumor cells contributing to cancer growth and metastasis. High VEGF expression has been described in lung cancer (Frezzetti et al., 2017). Ginsenoside Rg3 has anti-angiogenic effects, which may be related to the fact that ginsenoside Rg3 reduces the expression of genes related to vascular genetics (Tang et al., 2018). In agreement with the previous meta-analysis, ginsenoside Rg3 combined with chemotherapy could effectively reduce VEGF levels in serum of advanced NSCLC patients (*p* = 0.02).

Systemic chemotherapy typically has very limited efficacy, along with severe systemic adverse effects, such as gastrointestinal reactions, hematological toxicity, liver and kidney injury and so on, which severely affect NSCLC patients' quality of life (Mangal et al., 2017; Islam et al., 2019). Patients with advanced NSCLC are usually forced to interrupt treatment because they can not tolerate the severe side effects, which greatly reduces the treatment outcome. Compared to the previous study (Xu et al., 2016), our article provided a comprehensive analysis of the side effects, and the results suggested that ginsenoside Rg3 combined with chemotherapy was effective in reducing the incidence of gastrointestinal reactions and also played a certain role in reducing other side effects, although there was no statistical difference. In conclusion, ginsenoside Rg3 was safe and effective as an adjuvant for chemotherapy.

Recently, many researchers had conducted *in vitro* experiments on the anti-NSCLC effects of ginsenoside Rg3. Liu et al. (2019) Reported that ginsenoside Rg3 could upregulate VRK1 expression and P53BP1 foci formation in response to DNA damage, thereby inhibiting the tumorigenesis and viability of cancer cells. Futhermore, ginsenoside Rg3 could enhance the anticancer activity of Gefitinib through increasing apoptosis and decreasing migration in NSCLC cell lines (Dai et al., 2019). Therefore, ginsenoside Rg3 can effectively improve the efficacy and reverse drug resistance, suggesting that it can be used as an adjuvant in clinical treatment to benefit NSCLC patients.

### Limitations

Several limitations are worthy of discussion; firstly, the participants in the selected researches were all Chinese which may not be sufficiently representative. More studies with diverse populations are looking forward to validate the generalizability of our findings. Secondly, the blinded presentation of some studies is too simple or even missing, thus affecting the overall quality of the literature. Therefore, we may need more rigorous clinical experimental design to evaluate the efficacy and safety of ginsenoside Rg3 combined with chemotherapy for advanced NSCLC patients in the future. Thirdly, as an important parameter for evaluating the prognosis of patients with advanced NSCLC, the 5-year survival rate was missing in the original studies we analyzed. However, these studies reported 1-year and 2-year survival rate, both of which are important prognostic evaluation indicators for patients with advanced NSCLC as well. Finally, the sample size of articles screened in this study was not large enough due to the limited number of studies on the combination of chemotherapy and ginsenoside Rg3. More studies with high quality are expected to further validate the efficacy and safety of ginsenoside Rg3 in combination with first-line chemotherapy in advanced NSCLC.

## Conclusion

Ginsenoside Rg3 can enhance drug efficacy and reduce drug-induced toxicity from chemotherapy. The efficacy and safety of ginsenoside Rg3 in combination with first-line chemotherapy was superior to that of single chemotherapy in patients with advanced NSCLC. These findings provide helpful information for clinicians, indicating that ginsenoside Rg3 can be used as an adjuvant to optimize the treatment of advanced NSCLC.

## Data Availability

The raw data supporting the conclusions of this article will be made available by the authors, without undue reservation.
