# Supplementary material for: The Efficacy of Ginsenoside Rg3 Combined with First-line Chemotherapy in the Treatment of Advanced Non-Small Cell Lung Cancer in China: A Systematic Review and Meta-Analysis of Randomized Clinical Trials
Source: Front Pharmacol. 2021 Mar 18;11:630825. doi: 10.3389/fphar.2020.630825 (PMC8012535; doi:10.3389/fphar.2020.630825)
Supplement: Supplementary file 1 [file datasheet1.docx]

Supplementary

metareg logrr accept thearpy year tool numbers, wsse(selogrr) bsest(reml) knapphartung

Meta-regression Number of obs = 19

REML estimate of between-study variance tau2 = .04136

% residual variation due to heterogeneity I-squared_res = 73.26%

Proportion of between-study variance explained Adj R-squared = -28.78%

Joint test for all covariates Model F(5,13) = 0.54

With Knapp-Hartung modification Prob > F = 0.7442

------------------------------------------------------------------------------

logrr | Coef. Std. Err. t P>|t| [95% Conf. Interval]

-------------+----------------------------------------------------------------

accept | -.0401816 .1578125 -0.25 0.803 -.3811147 .3007515

thearpy | -.0645274 .0565967 -1.14 0.275 -.1867972 .0577423

year | -.1158649 .115888 -1.00 0.336 -.3662256 .1344958

tool | .021324 .0999486 0.21 0.834 -.1946018 .2372499

numbers | -.1799416 .204072 -0.88 0.394 -.6208123 .260929

_cons | .5752876 .2556762 2.25 0.042 .0229327 1.127643

**FIGURE S1.** The regression result of ginsenosides Rg3-containing chemotherapy on DCR.

A


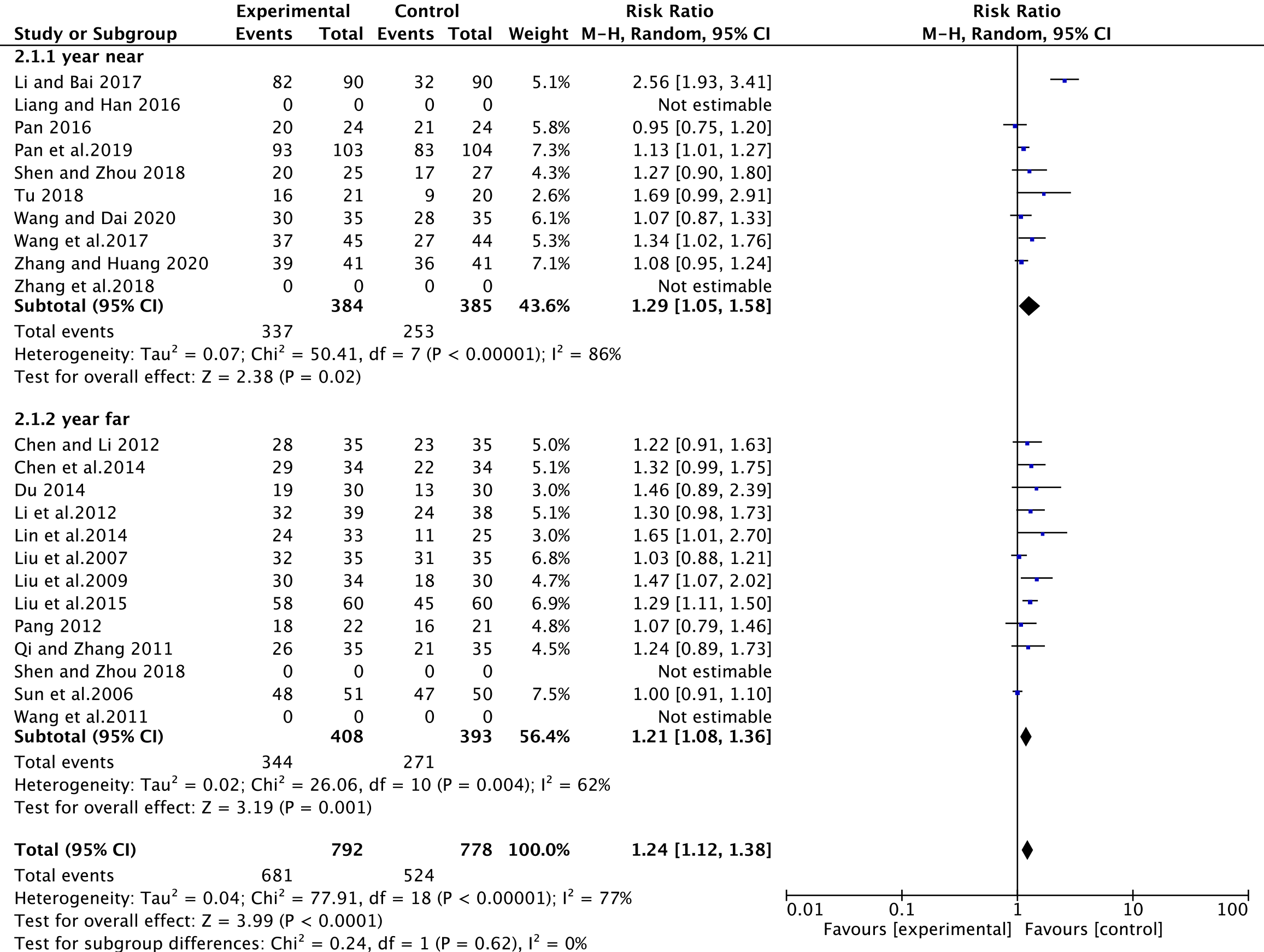


B


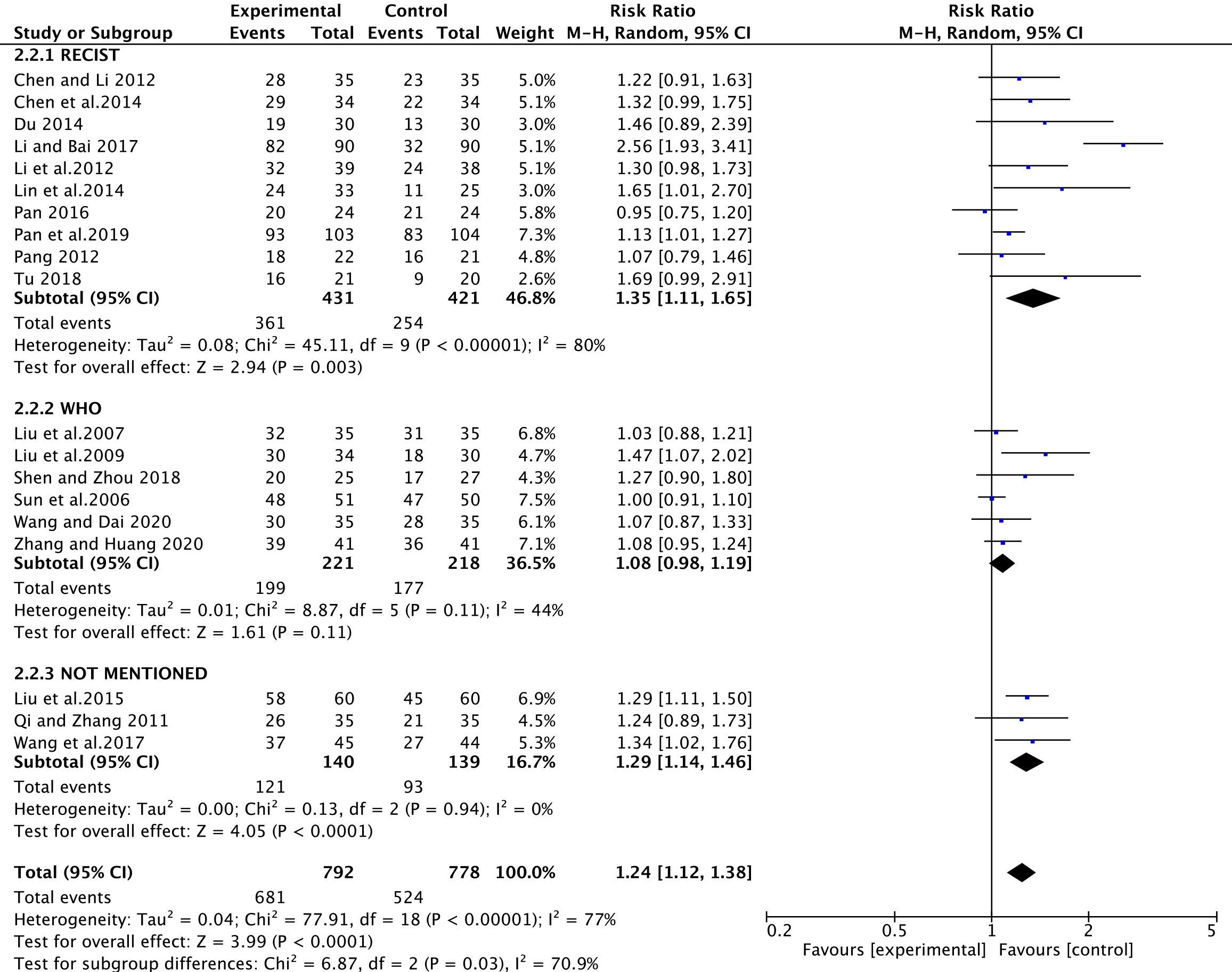


C
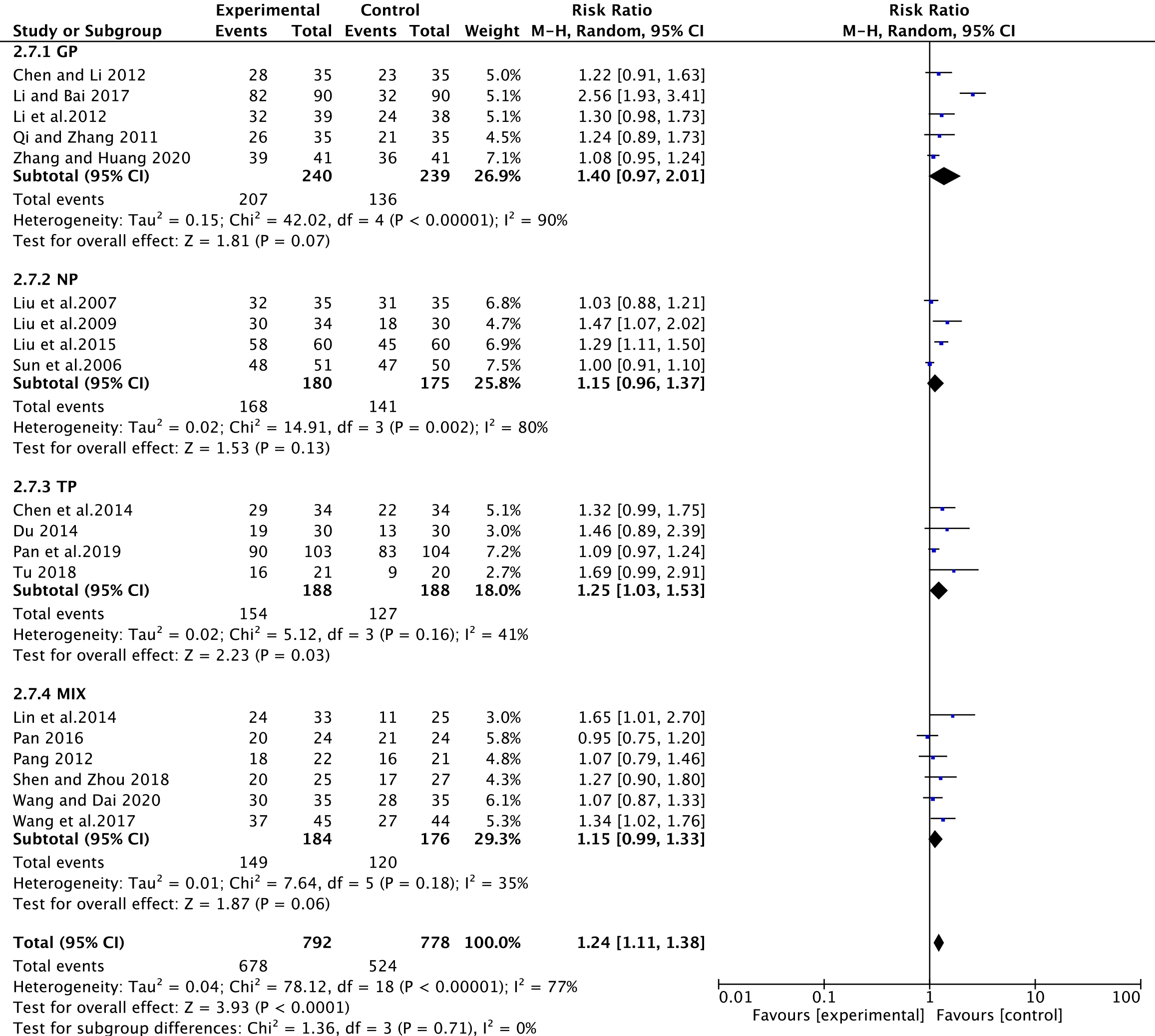


D
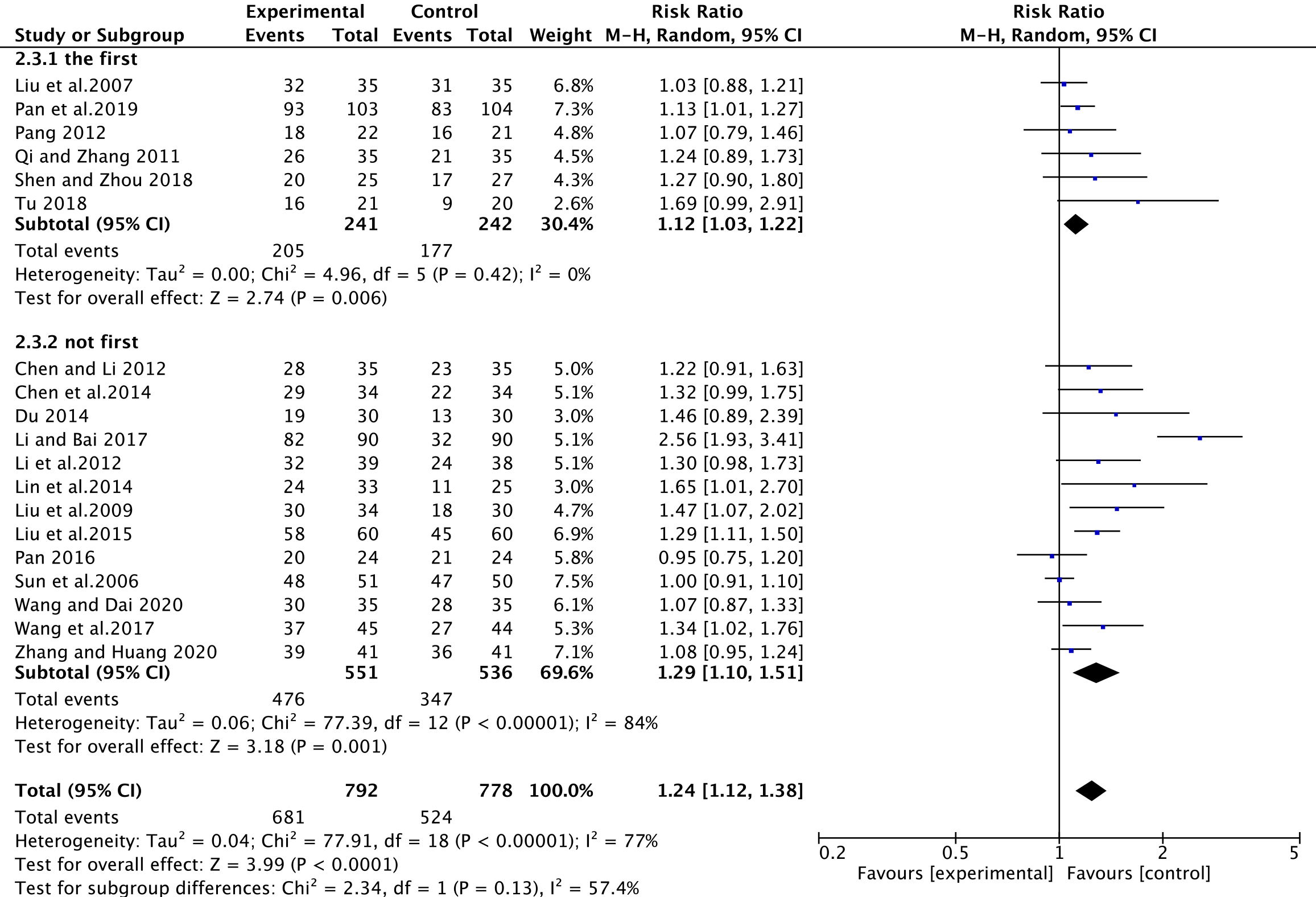


**FIGURE S2.** The subgroup analyses about (A) the publication year; (B) the evaluation criteria; (C) the drugs; (D) the first-treatment on DCR.


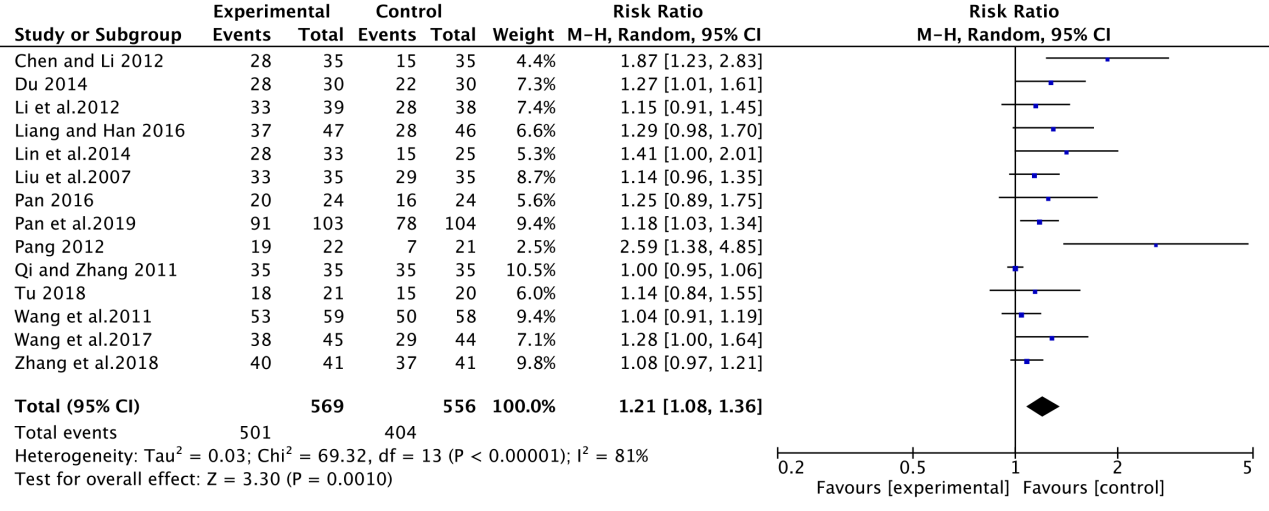


**FIGURE S3.** The pooled effects of ginsenosides Rg3-containing chemotherapy on KPS stabilization.


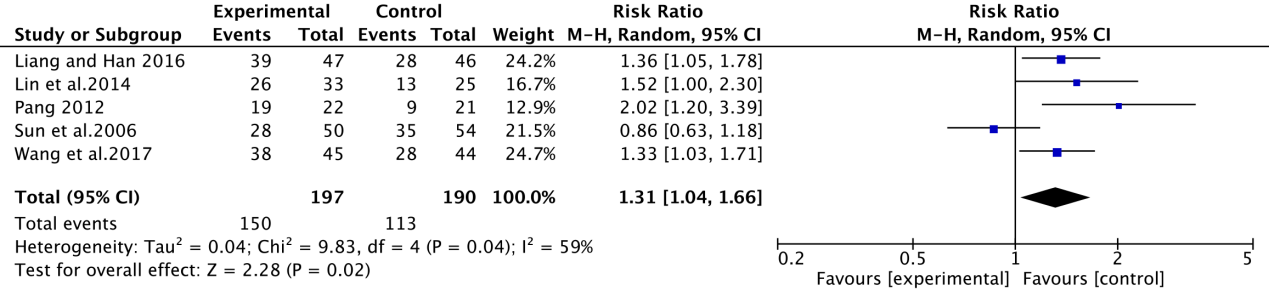


**FIGURE S4.** The pooled effects of ginsenosides Rg3-containing chemotherapy on weight change.


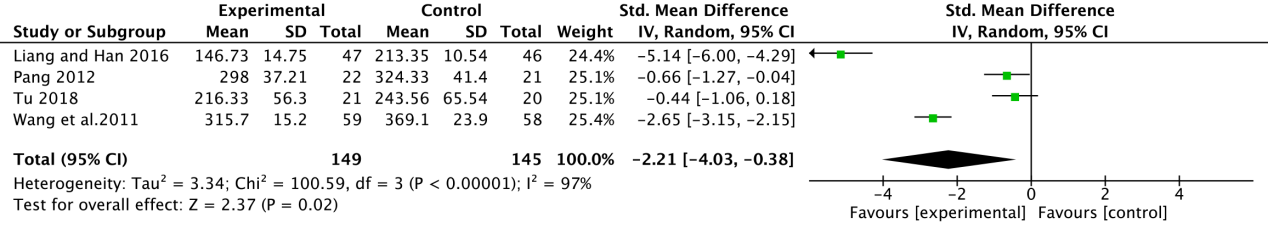


**FIGURE S5.** The pooled effects of ginsenosides Rg3-containing chemotherapy on VEGF leave.


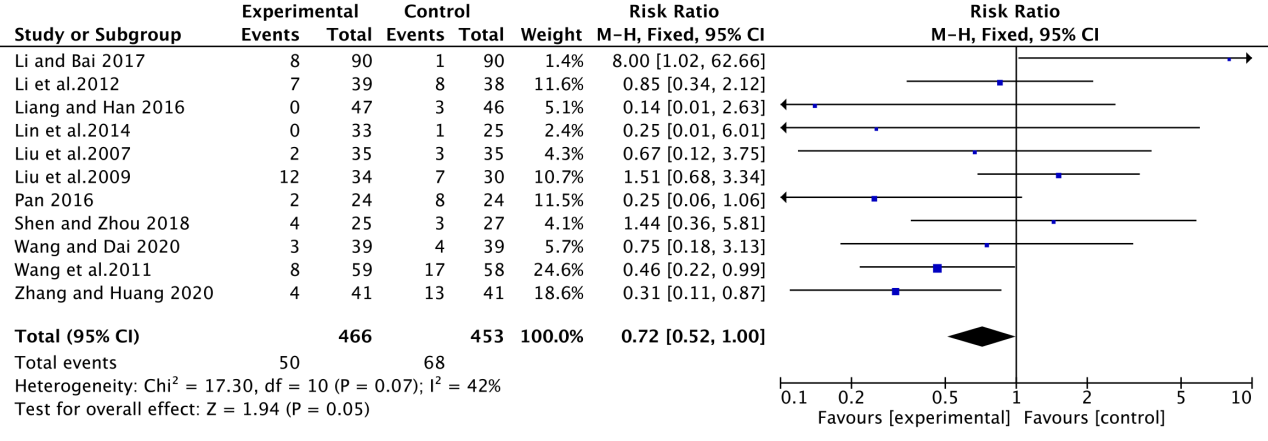


**FIGURE S6.** The pooled effects of ginsenosides Rg3-containing chemotherapy on liver and kidney abnormalities.


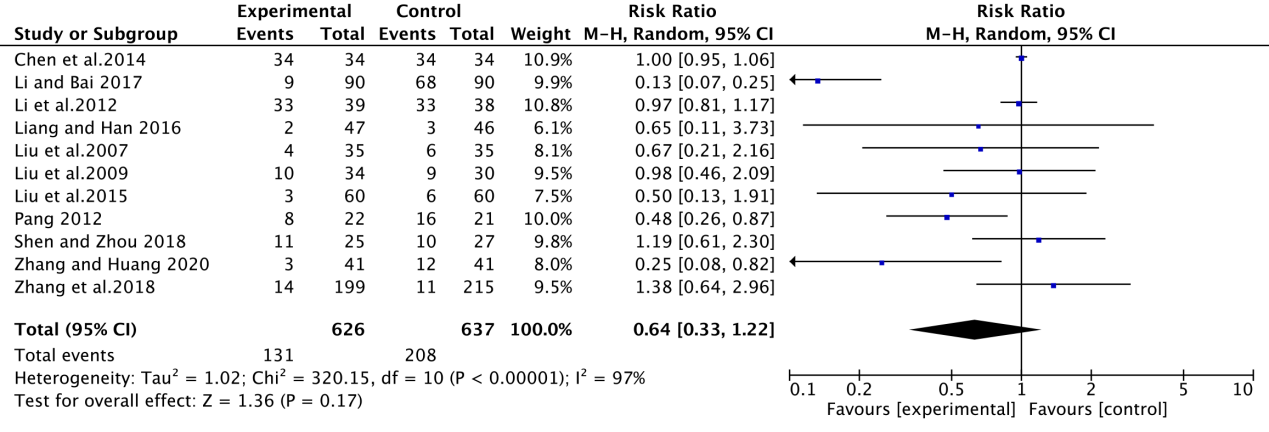


**FIGURE S7.** The pooled effects of ginsenosides Rg3-containing chemotherapy on thrombocytopenia.


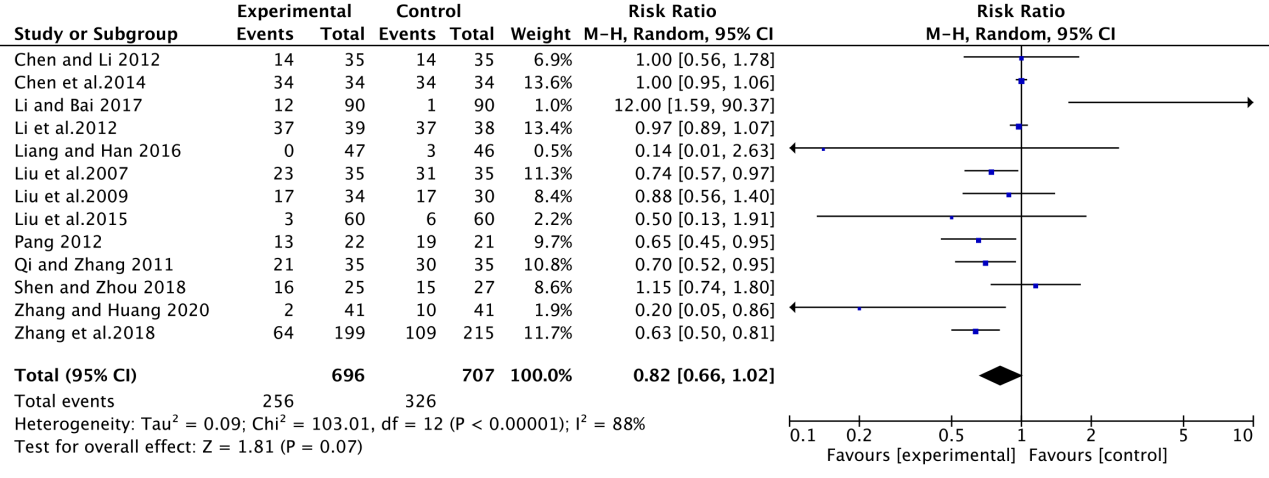


**FIGURE S8.** The pooled effects of ginsenosides Rg3-containing chemotherapy on leukopenia.


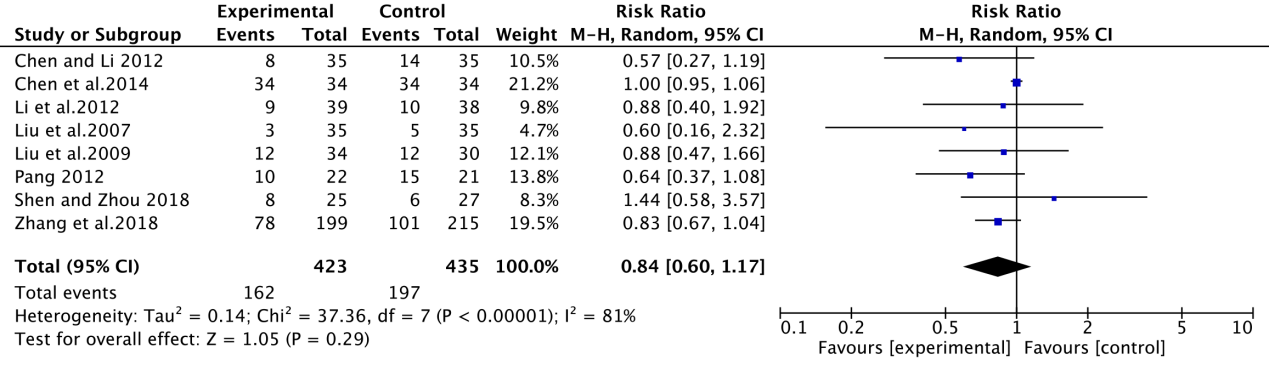


**Figure S9.** The pooled effects of ginsenosides Rg3-containing chemotherapy on reduced hemoglobin.


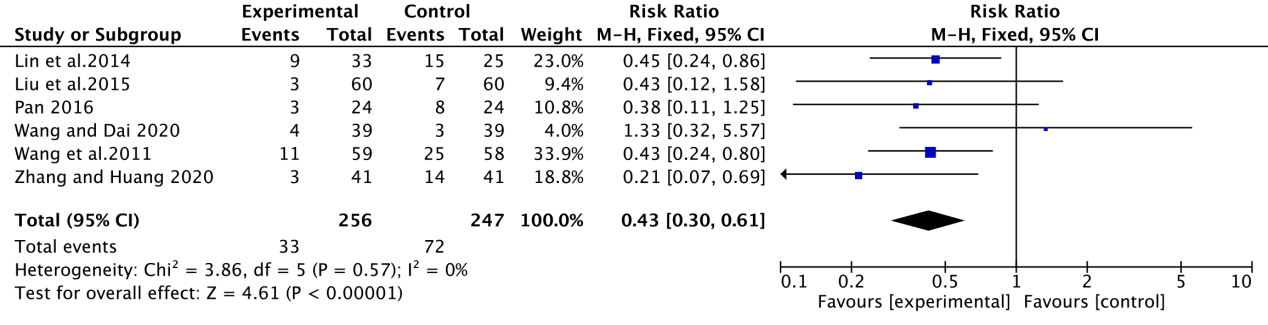


**Figure S10.** The pooled effects of ginsenosides Rg3-containing chemotherapy on bone marrow suppression.


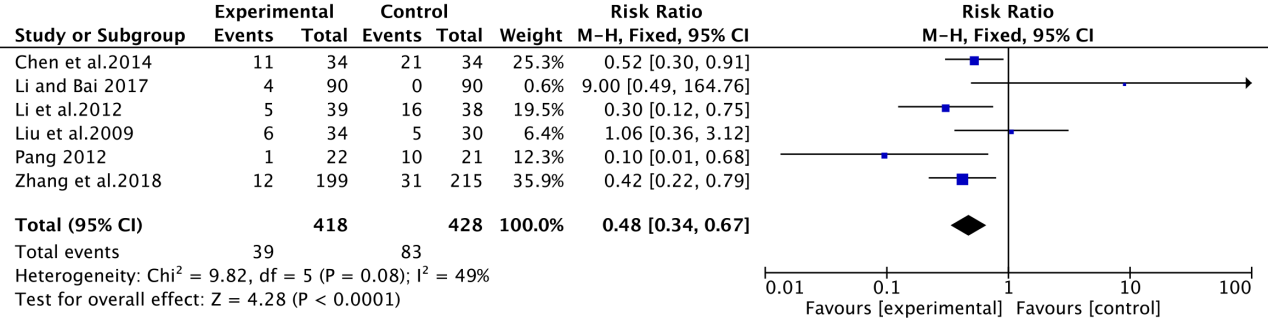


**Figure S11.** The pooled effects of ginsenosides Rg3-containing chemotherapy on leukopenia according to grade III-IV.
